# Idasanutlin and navitoclax induce synergistic apoptotic cell death in T-cell acute lymphoblastic leukemia

**DOI:** 10.1038/s41375-023-02057-x

**Published:** 2023-10-14

**Authors:** Kimberly B. Johansson, Megan S. Zimmerman, Iryna V. Dmytrenko, Feng Gao, Daniel C. Link

**Affiliations:** 1 Division of Oncology, Department of Medicine, Washington University School of Medicine, St. Louis, MO USA; 2 Medical Scientist Training Program, Washington University School of Medicine, St. Louis, MO USA; 3 Department of Pediatrics, Washington University School of Medicine, St. Louis, MO USA; 4 Department of Surgery, Washington University School of Medicine, St. Louis, MO USA

**Keywords:** Acute lymphocytic leukaemia, Acute lymphocytic leukaemia, Chemotherapy

## Abstract

T-cell acute lymphoblastic leukemia (T-ALL) is an aggressive hematologic malignancy in which activating mutations in the Notch pathway are thought to contribute to transformation, in part, by activating c-Myc. Increased c-Myc expression induces oncogenic stress that can trigger apoptosis through the MDM2-p53 tumor suppressor pathway. Since the great majority of T-ALL cases carry inactivating mutations upstream in this pathway but maintain wildtype *MDM2* and *TP53*, we hypothesized that T-ALL would be selectively sensitive to MDM2 inhibition. Treatment with idasanutlin, an MDM2 inhibitor, induced only modest apoptosis in T-ALL cells but upregulated the pro-apoptotic BH3 domain genes *BAX* and *BBC3*, prompting us to evaluate the combination of idasanutlin with BH3 mimetics. Combination treatment with idasanutlin and navitoclax, a potent Bcl-2/Bcl-xL inhibitor, induces more consistent and potent synergistic killing of T-ALL PDX lines in vitro than venetoclax, a Bcl-2 specific inhibitor. Moreover, a marked synergic response to combination treatment with idasanutlin and navitoclax was seen in vivo in all four T-ALL xenografts tested, with a significant increase in overall survival in the combination treatment group. Collectively, these preclinical data show that the combination of idasanutlin and navitoclax is highly active in T-ALL and may merit consideration in the clinical setting.

## Introduction

There is an unmet need for improved therapies to treat T-cell acute lymphoblastic leukemia (T-ALL), given the high rate of relapse and poor outcomes, especially in adults [1, 2]. Activating *NOTCH1* mutations are present in 60–70% of T-ALL cases [3], with inactivating mutations in *FBXW7*, a negative regulator of Notch signaling, present in an additional 15% of patients [4–8]. Although activation of Notch signaling and its downstream target c-Myc can trigger apoptosis through the ARF-MDM2-p53 tumor suppressor pathway, apoptosis is frequently inactivated in T-ALL through homozygous deletions of the *CDKN2A* locus encoding ARF in 80% of patients [9]. ARF antagonizes the function of MDM2, a nuclear-localized E3 ubiquitin ligase which targets p53 for degradation [10]. Inhibition of MDM2 ultimately leads to p53-mediated growth suppression and/or apoptosis [11]. Importantly, wildtype p53 expression is retained in more than 95% of primary T-ALL cases and 75% of relapsed cases [12, 13]. Based on these genomic data, we hypothesized that restoring the p19(ARF)-MDM2-p53 tumor suppressor pathway through MDM2 inhibition would increase p53 expression and lead to apoptosis in T-ALL.

In this study, we show that idasanutlin, a second generation small molecule inhibitor of MDM2, induces expression of pro-death BH3 domain family members *BAX* and *BBC3* (PUMA) but only modest apoptosis. Based on these observations, we hypothesized that inhibition of anti-apoptotic proteins would be synergistic with MDM2 inhibition in T-ALL. Navitoclax is an orally available investigational small molecule inhibitor of Bcl-2 family proteins [14]. Navitoclax acts as a BH3 mimetic within the BH3-binding domain of Bcl-2 anti-apoptotic proteins [15]. As T-ALL response to navitoclax has been transient [16, 17], we hypothesized improved efficacy using a combination of navitoclax and idasanutlin. For these studies, we used a panel of T-ALL PDX lines with the goal to recapitulate the most frequent genomic alterations in T-ALL and also include cases of early T-cell precursor ALL (ETP-ALL), an important leukemia subtype with distinct genomics and very poor clinical prognosis. We demonstrate that combination of BH3 and MDM2 inhibition acts synergistically to kill T-ALL cell lines and patient-derived xenografts (PDX) both in vitro and in vivo.

## Materials and methods

### T-ALL cell lines and PDX cells

The human T-ALL MOLT-3 cell line was previously kindly provided by the lab of Dr. Grant Challen (Washington University School of Medicine, St. Louis, MO, USA) [18]. MOLT-3 cells were maintained in ATCC-formulated RPMI-1640 Medium containing 10% heat-inactivated fetal bovine serum with 100 U/ml penicillin and streptomycin (#15140122; Gibco) at 37 °C with 5% CO_2_ in a humidified incubator. The mouse T-ALL PDX lines DFCI12, DFCI15, DFAT28537, DFAT27681, and CBAT27299 were previously established and kindly provided by the cBioPortal (Dana Farber Cancer Institute, Boston, MA, USA) [19, 20]. The PDX cells were maintained in StemSpan^TM^ SFEM II (#9655, Stem Cell Technologies), penicillin-streptomycin (100 U/ml), human IL-2 (0.2 IU/ml), human IL-7 (10 ng/ml), and human stem cell factor (50 ng/ml).

### CRISPR/Cas9 knockout

For generation of knockout cells using the clustered regularly interspaced short palindromic repeats (CRISPR)/Cas9 system, single-guide RNA (sgRNAs) targeting *TP53* or the AAVS1 safe-harbor locus were purchased (Integrated DNA Technologies) and mixed with recombinant Cas9 protein (IDT), incubated at room temperature to generate ribonucleoprotein complexes, and transferred into MOLT-3 cells using the Neon transfection system at 1350 V, 35 ms, 1 pulse, following manufacturer recommendations. Isogenic clones were generated through outgrowth following single cell sorting. Insertion/deletion status was assessed by next-generation sequencing of the target locus on the Illumina MiSeq platform and analyzed using CRISPResso2 [21], and knockout was confirmed through immunoblotting 6 h following x-ray irradiation (10 Gray, KUBTEC XCELL 50). Cell lines are monitored with STR profiling and mycoplasma testing.

### Quantitative real-time PCR and immunoblotting

The methods followed for real-time PCR and immunoblotting were as previously described in [22] and are detailed in the Supplementary Methods, including specific reagents and antibodies.

### Proliferation and caspase activity assays

T-ALL cell lines and PDX cells were subjected to CellTiter-Glo assay (#PRG9242; Promega) and Caspase-Glo 3/7 assay (#PRG8091; Promega) as detailed in the Supplementary Methods.

### Flow cytometry

T-ALL cell lines and PDX cells were subjected to flow cytometry assessment of cell cycle, apoptosis, and viability as previously described in [23] and are detailed in the Supplementary Methods. In vivo PDX models underwent peripheral blood flow cytometry for human CD45.

### In vivo PDX models of T-ALL

All animal studies were approved by the Institutional Animal Care and Use Committee at Washington University in St. Louis under protocol number 20-0495. DFCI12, DFCI15, DFAT28537, and CBAT27299 PDX cells were engrafted into NSG mice and treated with vehicle alone, idasanutlin, and/or navitoclax with hCD45 flow cytometry as described in the Supplementary Methods. Upon evidence of engraftment, mice were stratified by degree of tumor burden, and mice from each tier were randomized equally into treatment cohorts. These random groups were treated with a proportional mixture of both vehicles, idasanutlin (RG7388, Roche investigational basis) (40 mg/kg by oral gavage daily on a 5-days-on 2-days-off schedule for 14 days), navitoclax (ABT-263, Chemgood C-1009 [24, 25]) (100 mg/kg by oral gavage daily for 14 days), or combination therapy. Idasanutlin was sonicated into suspension in Roche GTX011795 solvent (Water 97.8% + Hydroxypropyl cellulose 2% + Polysorbate 80 0.1% + Methyl paraben 0.09% + Propyl paraben 0.01% + Sodium acetate 0.0072% + Acetic acid, glacial 0.0569%). Navitoclax was formulated per manufacturer’s instructions by sonicating into suspension in 60% phosal 50 propylene glycol, 30% polyethylene glycol 400, and 10% ethanol. Two independent experiments were performed with cohort sizes ranging from 15 (3–4 per treatment) to 31 (7–8 per treatment), for a pooled minimum of *n* = 10 per treatment, per PDX line. Minimum cohort size determined based on estimated effect size following pilot experiment. Based on the law of diminishing returns [26] recommended that a degree of freedom (DF) of 10–20 associated with error term in an analysis of variance (ANOVA) would be adequate to estimate preliminary information with a good precision.

### Statistical analyses

Statistical testing was performed using Prism (version 9, GraphPad Software). The between-group differences in tumor burden were compared using unpaired Student’s *t* test for independent samples or two-way analysis of variance for repeated measurement data. Model diagnosis was performed graphically based on residuals from each model. The differences in survival were summarized using Kaplan–Meier curves and compared by log-rank tests. Synergy was calculated in vitro using SynergyFinder 2.0 to determine a ZIP (zero interaction potency) score [27, 28]. Synergy was calculated for in vivo studies using a modified Bliss Independence test, analyzing average daily change between the dual therapy and the most effective single agent therapy. All experiments were performed with at least two independent experiments, each in triplicate, unless otherwise noted. Sample size is specified in each panel with all data points plotted individually for *n* < 5. All data are presented as mean ± standard error of the mean. A *p* value of <0.05 was considered significant.

## Results

### Idasanutlin has p53-dependent activity against T-ALL cells

We hypothesized that *TP53*-sufficient T-ALL is sensitive to MDM2 inhibition. To test this hypothesis, we initially studied MOLT-3 cells, an immortalized T-ALL cell line with wildtype *TP53*, and generated isogenic cell lines edited at *TP53* or the AAVS1 safe-harbor locus (control). After identifying frameshift indels in each allele by sequencing, we validated p53 loss by assessing protein level following x-ray irradiation. We observed strong induction of p53 protein in the AAVS1-edited control lines, but completely absent p53 in both *TP53* knockout (KO) lines tested, each of which carried different indel mutation combinations (Fig. S1A).

**Fig. 1:**
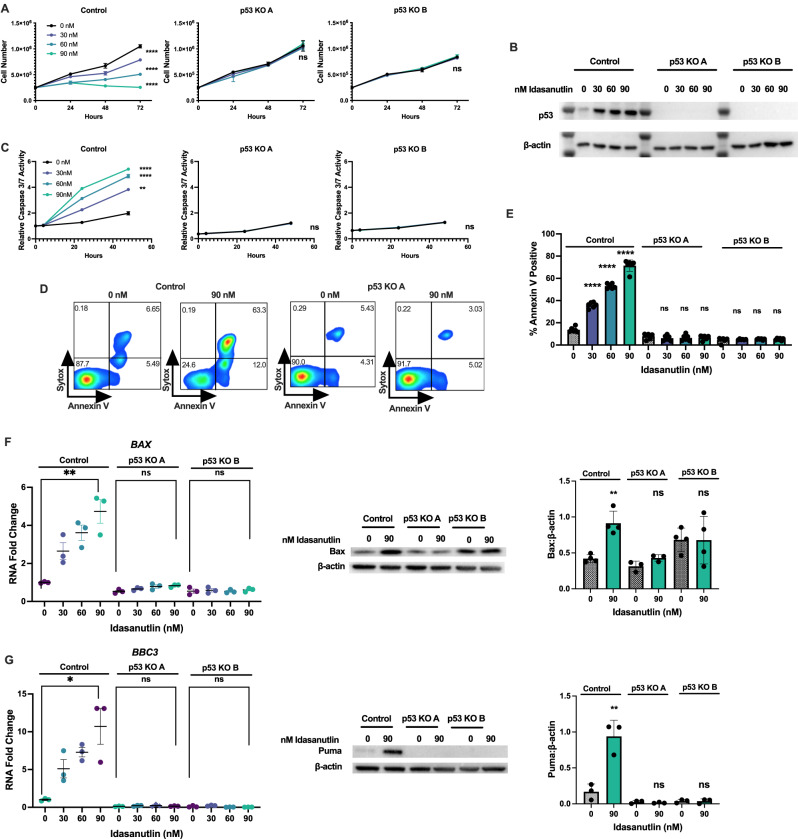
Idasanutlin has p53-dependent activity against T-ALL immortalized cell lines. **A** Cell number of isogenic AAVS-1 targeted (control) or *TP53*^*−/−*^ (p53 KO) MOLT-3 cells cultured with idasanutlin (30, 60, 90 nM) over 72 h. **B** Protein levels of p53 and *β*-actin in MOLT-3 control or p53 KO lines after 24 h of idasanutlin treatment. **C** Relative caspase-3/7 activity over 48 h of vehicle or idasanutlin treatment. **D** Representative Annexin V apoptosis and Sytox viability staining of MOLT-3 control (left) or p53 KO (right) cells following 48 h of treatment. **E** Percent apoptotic cells measured by Annexin V positivity following 48 h of treatment. **F** Expression levels of p53 target gene *BAX* RNA (left) and Bax protein (center representative immunoblot, right quantification relative to *β*-actin) in MOLT-3 cell lines after 24 h of idasanutlin treatment. **G** Expression levels of p53 target gene *BBC3* RNA (left) and Puma protein (center representative immunoblot, right quantification relative to *β*-actin) in MOLT-3 cell lines after 24 h of idasanutlin treatment. All experiments performed in triplicate with at least two independent experiments. Error bars represent SEM (**** ≤ 0.0001, *** ≤ 0.001, ** ≤ 0.01, * ≤ 0.05 compared to DMSO). **A**, **C** Two-way ANOVA, **E**–**G** unpaired *t*-test.

MOLT-3 control cells exhibited a dose-dependent sensitivity to idasanutlin, whereas both p53 KO cell lines were resistant (Fig. 1A). As predicted, treatment with idasanutlin resulted in stabilization and accumulation of p53 protein in only the control line (Fig. 1B). Ultimately, idasanutlin treatment induced apoptosis of MOLT-3 control cells but not the p53 knockout lines, as evidenced by increased caspase-3/7 activity (Fig. 1C) and by cell surface annexin-V expression (Fig. 1D, E). Idasanutlin treatment also resulted in strong induction of the pro-apoptotic p53 target genes *BBC3* and *BAX* at both the RNA and protein level, which was lost in p53 knockout cells (Fig. 1F, G). In order to confirm MDM2 as a relevant target, we also treated cells with the chemically distinct MDM2 inhibitor MI-773 (SAR405838). Treatment with this alternative compound induced a p53-dependent decrease in cell number (Fig. S1B) as well as induction of apoptosis as indicated by increased activated caspase-3/7 limited to the control line (Fig. S1C).

**Fig. 2:**
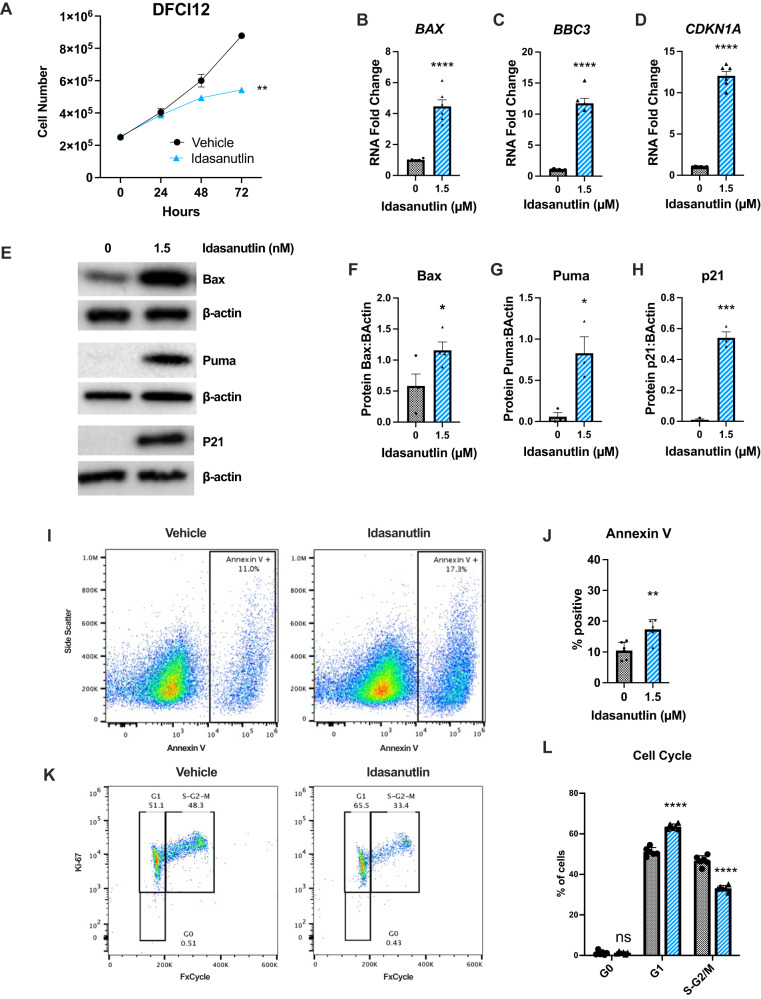
Idasanutlin has modest in vitro activity against a T-ALL PDX line. **A** Cell number of the DFCI12 cells over 72 h of treatment with idasanutlin (1.5 μM) or vehicle control. Expression of *BAX* (**B**), *BBC3* (**C**), and *CDKN2A* (**D**) mRNA 24 h into treatment; data are normalized to *β*-actin. **E** Representative immunoblots for Bax, Puma, p21 and *β*-actin following 24 h of treatment. Quantification of protein levels of Bax (**F**), Puma (**G**), and p21 (**H**) relative to *β*-actin at 24 h. **I** Representative flow cytometry plots for Annexin V staining of vehicle (left) and idasanutlin (right) cells following 48 h of treatment. **J** Percent apoptotic cells measured by Annexin V positivity following 48 h of treatment. **K** Representative flow cytometry distribution for FxCycle Ki-67 staining of vehicle (left) and idasanutlin (right) following 48 h of treatment. **L** Cell cycle plots following 48 h of treatment. All experiments performed in triplicate with at least two independent experiments. Error bars represent SEM (**** ≤ 0.0001, *** ≤ 0.001, ** ≤ 0.01, * ≤ 0.05 compared to DMSO). **A** Two-way ANOVA, **B**–**D**, **F**, **G**, **J**, **L** unpaired *t*-test.

We next assessed the response of DFCI12, a T-ALL PDX, to idasanutlin. DFCI12 cells carry an activating *NOTCH1* mutation but are wildtype for *TP53* (Table 1). DFCI12 cells were sensitive to 1.5 μM idasanutlin, a higher dose than that required for MOLT-3 cells, but within the range of idasanutlin achieved in the serum in vivo (Fig. 2A) [29, 30]. In DFCI12 cells, treatment with idasanutlin induced expression of the *TP53* target genes *BAX*, *BBC3* (Puma), and *CDKN1A* (p21) at the RNA (Fig. 2B–D) and protein levels (Fig. 2E–H), and resulted in a modest increase in apoptosis (Fig. 2I, J). Consistent with increased *CDKN1A* expression, idasanutlin treatment induced a modest, but significant, decrease in cycling cells, with an increase in cells in the G1 phase and a decrease in cells in the S-G2-M phases of the cell cycle (Fig. 2K, L). Together, these data suggest that MDM2 inhibition has modest activity against T-ALL.

**Table 1:** T-ALL PDX selected clinical and molecular characteristics.

Name	WHO classification	Sex	Age	NOTCH1	FBXW7	CDKN2A	TP53	Cytogenetics	Treatment phase when sampled
DFCI12	T-ALL	M	16.11	L1574P, T432M			Wildtype	47XY,+8,add(9)(p13)	Unknown
DFCI15	T-ALL	M	6.11	F1592S, P2514X		Deleted	Wildtype	46XY	Untreated
DFAT-28537	T-ALL	M	25	F1592S	R465H	Deleted	Wildtype	Unknown	Untreated
DFAT-27681	T-ALL ETP	M	23			Deleted	Wildtype	46,XY,t(1;12)(q21;p13)	Relapse post-allogeneic HSCT
CBAT-27299	T-ALL ETP	M	5				Wildtype	46XY	Untreated

### Idasanutlin and navitoclax demonstrate synergistic activity against T-ALL PDX lines

**Fig. 3:**
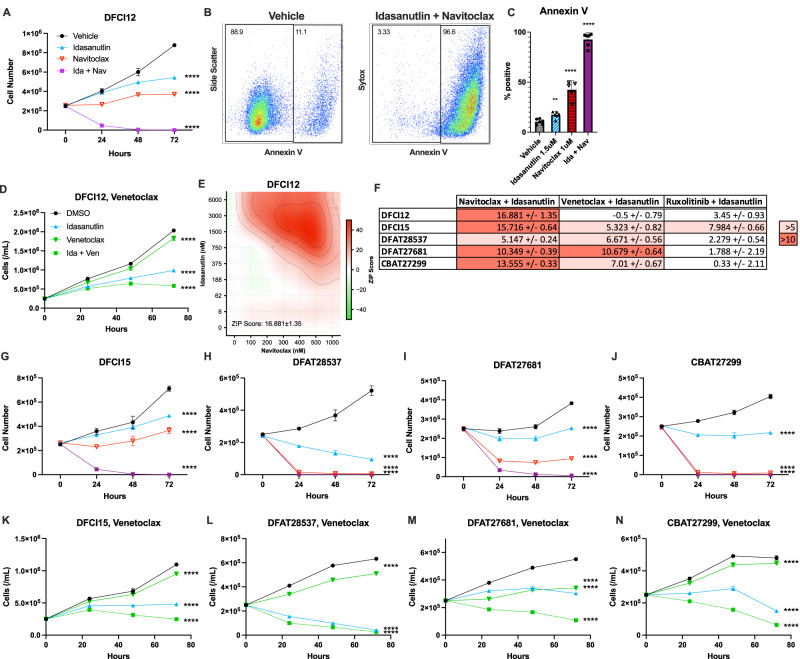
The combination of idasanutlin and navitoclax has synergic activity against T-ALL PDX lines in vitro. **A** DFCI12 cells were treated in vitro over 72 h with vehicle, idasanutlin (1.5 μM), navitoclax (1 μM), or combination therapy and cell number quantified. **B** Representative flow plot for Annexin V staining of vehicle (left) and dual-treated (right) cells following 48 h of treatment. **C** Percent apoptotic cells by Annexin V positivity following 48 h of treatment. **D** DFCI12 cells were treated in vitro over 72 h with vehicle, idasanutlin (1.5 μM), venetoclax (1 μM), or combination therapy and cell number was quantified. **E** DFCI12 cells were treated with increasing doses of idasanutlin (vehicle, 6 nM, 60 nM, 188 nM, 375 nM, 750 nM, 1.5 μM, 3 μM, 6 μM) and navitoclax (vehicle, 100 nM, 200 nM, 300 nM, 500 nM, 500 nM, 1 μM) to generate a dose-response matrix and evaluate for ZIP synergy score with SynergyFinder 2.0. Representative panel from two independent experiments. **F** Zip synergy scores for the indicated T-ALL PDX line based on dose-response matrix data testing the indicated drug combination. Primary data are presented in Figs. S2–4. DFCI15 (**G**), DFAT28537 (**H**), DFAT27681 (**I**), or CBAT27299 (**J**) cells were treated in vitro over 72 h with vehicle, idasanutlin (1.5 μM), navitoclax (1 μM), or combination therapy and cell number quantified. DFCI15 (**K**), DFAT28537 (**L**), DFAT27681 (**M**), or CBAT27299 (**N**) cells were treated in vitro over 72 h with vehicle, idasanutlin (1.5 μM), venetoclax (1 μM), or combination therapy and cell number quantified. All experiments performed in triplicate with at least two independent experiments. Error bars represent SEM (**** ≤ 0.0001, *** ≤ 0.001, ** ≤ 0.01, * ≤ 0.05 compared to DMSO). **A** Two-way ANOVA, **C** unpaired *t-*test, **D** two-way ANOVA, **E**, **F** Zero Interaction Potency Score, **G**–**N** two-way ANOVA.

The induction of expression of pro-death BH3 domain family members *BAX* and *BBC3* suggested that MDM2 inhibition may prime T-ALL cells for induction of apoptosis after treatment with inhibitors of pro-survival BH3 domain proteins, such as venetoclax (targeting Bcl-2) or navitoclax (targeting Bcl-2 and Bcl-xL). Since a prior study showed increased activity of navitoclax compared to venetoclax in T-ALL [31], we next assessed the response of DFCI12 cells to idasanutlin alone, navitoclax alone, or the combination. Whereas treatment with navitoclax or idasanutlin alone modestly inhibited DFCI12 cell growth, combination therapy resulted in complete killing (Fig. 3A). Indeed, combination therapy strongly induced apoptosis, with nearly all cells after 24 h showing Annexin V cell surface staining (Fig. 3B, C). A formal dose-response matrix was generated to assess the synergy of the drug combination in T-ALL cell killing (Fig. 3E). Synergy was assessed by ZIP (zero interaction potency), a statistical method developed by Yadav et al. that compares the change in potency of individual dose response curves in the presence of a second agent [27]. Generally, a ZIP score greater than 10 is considered evidence for synergy. Idasanutlin and navitoclax combination therapy had an overall ZIP synergy score of 16.9 ± 1.35. The most synergistic area scores of up to 51.8 were observed at 0.75–6 uM idasanutlin and 0.3–1 uM navitoclax, which are clinically achievable drug concentrations.

To determine whether the sensitivity to idasanutlin and navitoclax dual therapy extended beyond DFCI12 cells, we treated a panel of four additional T-ALL PDX lines in vitro with vehicle alone, idasanutlin alone, navitoclax alone, or dual therapy. Including DFCI12 cells, the five different PDX lines represent a range of T-ALL genetics (Table 1). *NOTCH1* and/or *FBXW7* mutations are present in three lines; *CDKN2A* biallelic loss is present in three lines; and all lines are wildtype for *TP53*. Two lines are ETP-ALL with wildtype *NOTCH1*, *FBXW7*, and *TP53*. For all five PDX lines tested, in vitro dual treatment led to robust cell death within 48 h. For two of the cell lines tested, in vitro treatment with 1 μM navitoclax alone was also highly efficacious (Fig. 3G–J). Additionally, we treated each of these lines with a formal dose-response matrix to assess potential synergic activity of idasanutlin and navitoclax in combination. We identified that of the five lines tested, one suggested an additive effect of the therapy and the remaining 4 demonstrated synergic activity with ZIP scores ranging from 10.3–16.9 (Figs. 3F and S2A–D). Additionally, while the MOLT-3 T-ALL control line was responsive to combination therapy, the p53 knockout MOLT-3 line did not demonstrate a response with a ZIP synergy score of −1.48 ± 2.61 (Fig. S2E, F), suggesting the synergic effect of therapy is p53-dependent.

Venetoclax is another BH3 mimetic of interest, which inhibits Bcl-2 but not Bcl-xL. Prior studies examining venetoclax in combination with MDM2 inhibition in the context of acute myeloid leukemia have shown promising results [32]. However, previous studies have shown that a majority of T-ALL samples have increased sensitivity to navitoclax compared to venetoclax [31, 33]. To directly assess the activity of venetoclax, we treated each T-ALL PDX line with venetoclax alone, idasanutlin alone, or the combination. At clinically relevant doses of venetoclax [34], we observed a statistically significant, but modest, growth inhibition with all 5 T-ALL PDX lines that was potentiated by idasanutlin (Fig. 3D, K–N). We next tested a dose-response matrix, which showed which showed clear synergy with idasanutlin in a single T-ALL PDX line (DFAT27681), with an additive effect in 3 lines, and no response in DFC12 cells (Figs. S3 and 3F).

There is evidence for increased JAK/STAT signaling in a subset of T-ALL [35, 36], with in vitro studies showing activity of JAK2 inhibitors alone or in combination with dexamethasone [37] or MDM2i [38] in *JAK3*-mutated T-ALL. To directly assess the activity of JAK2 inhibitors, we treated the T-ALL PDX lines with ruxolitinib (a competitive inhibitor of the JAK1 and JAK2 kinases) alone, idasanutlin alone, or the combination. At clinically relevant doses [39, 40], ruxolitinib treatment induced a statistically significant, but modest, growth suppression in all T-ALL PDX lines, which was potentiated by the addition of idasanutlin (Fig. S4A–E). However, analysis of dose-response matrix data showed no strong synergic effect for the combination of ruxolitinib and idasanutlin in any of the five T-ALL PDX lines tested (Figs. S4F–J and 3F). Collectively, these data suggest that the combination of navitoclax plus idasanutlin provides more consistent and potent synergistic killing of T-ALL PDX lines than the combination of venetoclax with idasanutlin or ruxolitinib with idasanutlin.

### Transcriptional characterization of treated PDX lines

**Fig. 4:**
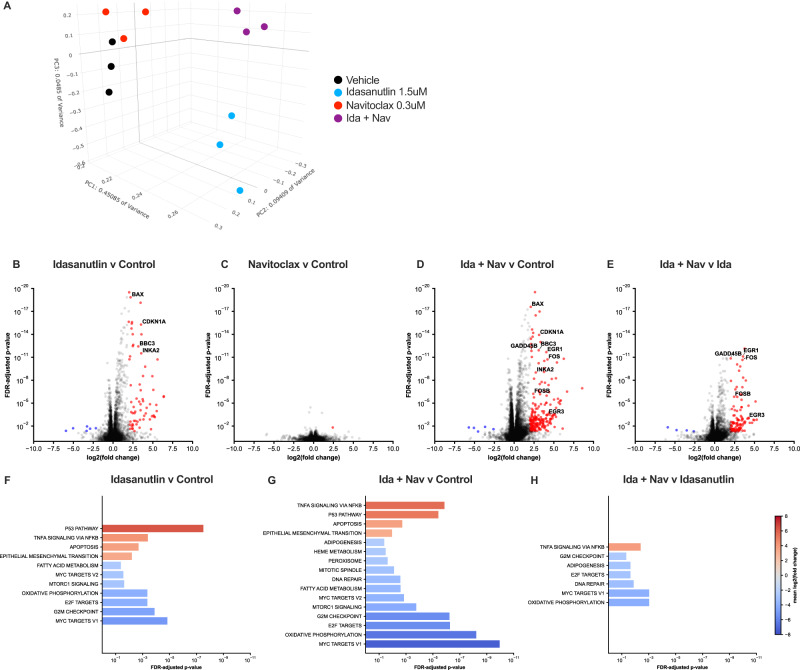
Transcriptional characterization of treated PDX lines. **A** Principal components analysis clustering of transcriptomes from vehicle only, idasanutlin (1.5 μM), navitoclax (300 nM), and combination therapy-treated cells. Differential expression of cells treated with: idasanutlin compared to vehicle (**B**), navitoclax compared to vehicle (**C**), combination compared to vehicle (**D**), or combination compared to idasanutlin only (**E**). Significantly downregulated genes are shown in blue and upregulated in red. Select p53-pathway genes of interest are annotated. All significant enriched Human Molecular Signatures Database (MSigDB) Hallmark pathways for idasanutlin compared to vehicle (**F**), combination compared to vehicle (**G**), and combination compared to idasanutlin only (**H**). No Hallmark pathways were significantly altered in navitoclax relative to vehicle. Three independent samples were analyzed for each condition. Genes are reported as significantly differentially expressed when FDR-adjusted *p* value ≥0.5 and log fold change ≥2 or ≤−2.

To explore mechanisms of synergy, we performed RNA sequencing on DFCI12 cells treated with idasanutlin alone, navitoclax alone, the combination, or vehicle-only for 16 h, when the majority (≥70%) of cells were viable and non-apoptotic as measured by Annexin-V staining (Fig. S5). Principal components analysis of these samples reveals a close clustering of navitoclax single treatment with control, while idasanutlin alone and dual therapy are each distinct (Fig. 4A). Accordingly, there was only one significantly differentially expressed gene induced by navitoclax treatment alone, which is consistent with navitoclax primarily acting post-transcriptionally to activate apoptosis (Fig. 4B and Table S1). Idasanutlin treatment led to the significant upregulation of 73 genes, including known p53 target and cellular stress response genes, such as *INKA2*, *BAX*, *BBC3*, and *CDKN1A* (Fig. 4C, D and Table S1). Indeed, gene set enrichment analysis identified the Hallmark p53 pathway as the top upregulated pathway in idasanutlin-treated cells (Fig. 4F, G). Finally, a total of 217 significantly differentially expressed genes were identified comparing idasanutlin with combination treated cells (Table S1). The top upregulated gene expression pathway in combination treated cells compared to idasanutlin alone was the Hallmark TNF*α* signaling via NF*κ*B (Fig. 4H). Indeed, marked increased expression of certain known NF*κ*B target genes was observed, including *EGR1*, *EGR3*, *FOS*, *FOSB*, and *GADD45B* (Fig. 4E).

### The combination of idasanutlin and navitoclax is highly active in human T-ALL xenotransplantation models

**Fig. 5:**
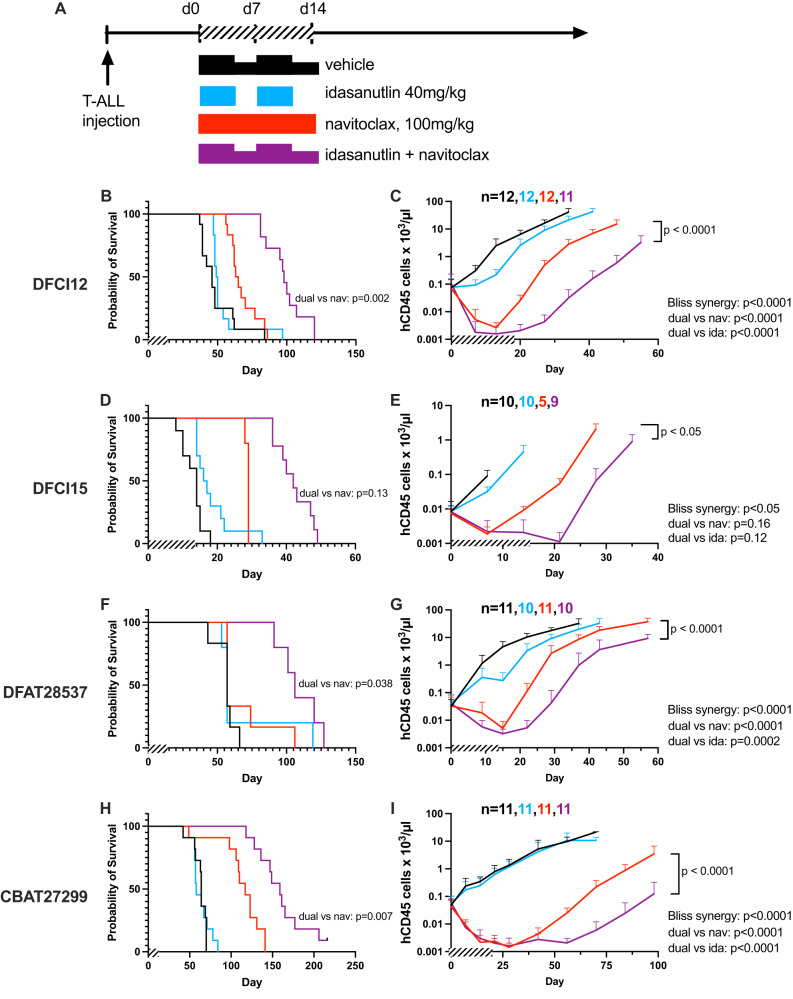
The combination of idasanutlin and navitoclax is highly active in human T-ALL xenotransplantation models. **A** Each human T-ALL xenograft was injected into NSG mice and allowed to engraft. Once engraftment of T-ALL was observed, mice were randomized to one of four treatments: (1) vehicle alone; (2) idasanutlin 40 mg/kg by oral gavage daily on a 5-days-on 2-days-off schedule for 14 days; (3) navitoclax 100 mg/kg by oral gavage daily for 14 days; (4) combined idasanutlin and navitoclax. Two independent experiments were performed with cohort sizes ranging from 15 (3–4 per treatment) to 31 (7–8 per treatment). Survival was tabulated and leukemic burden was measured in the blood by flow cytometry for human CD45+ cells for the DFCI12 (**B**, **C**), DFCI15 (**D**, **E**), DFAT28537 (**F**, **G**), and CBAT27299 (**H**, **I**) lines. Two independent experiments were performed per line, with cohort size minimum *n* = 10. Error bars represent SEM (**** ≤ 0.0001, *** ≤ 0.001, ** ≤ 0.01, * ≤ 0.05 compared to vehicle). Variance in tumor burden was similar between the treatment groups. **B**, **D**, **F**, **H** Pairwise comparisons, **C**, **E**, **G**, **I** Synergy assessed with modified Bliss Independence test, analyzing average daily change in tumor burden for synergy interaction.

Having observed strong in vitro activity against a panel of T-ALL PDX lines, we next assessed the in vivo response of four different T-ALL xenografts to dual idasanutlin and navitoclax treatment. For each line, human T-ALL cells were injected into immunodeficient mice, and following engraftment, mice were randomized into treatment groups. Mice were treated for 14 days before withdrawal of therapy, and tumor burden as well as survival were analyzed (Fig. 5A). Combination therapy was tolerated in the mice, with minor transient weight loss observed.

Treatment with navitoclax induced a significant decrease in T-ALL burden in all four xenotransplants (Fig. 5B–I). Treatment with idasanutlin alone induced a significant decrease in T-ALL burden in 3 of 4; the non-responding xenograft was an ETP-ALL (Fig. 5H, I). Finally, a marked response to combination treatment with idasanutlin and navitoclax was seen in all four T-ALL xenografts, exceeding a predicted additive effect based on monotherapy response. This combination was found to be synergistic in each case based on a modified Bliss Independence test, analyzing average daily change in tumor burden for synergy interaction. In addition, overall survival was significantly increased in the combination treatment group.

## Discussion

T-ALL is an aggressive hematologic malignancy that comprises 15% of pediatric ALL and 25% of adult ALL [3, 41]. Current treatment consists of intense chemotherapy that is associated with acute and chronic life-threatening or debilitating toxicities [42]. Five-year event-free survival is 70–75% for children, 30–40% for adults under 60, and less than 10% for adults over age 60 [43]. The prognosis after relapse is dismal, with 3-year event-free survival of only 10–15% [1, 44–46]. Allogeneic hematopoietic cell transplantation (HCT) may be curative for relapsed T-ALL; however, in the largest study performed to date, overall survival was only 24% after a median follow-up of 2 years [47]. Thus, there remains an unmet clinical need for better T-ALL therapeutics. The present study explores the feasibility of reactivating oncogenic stress sensing and the consequent apoptotic response through combined MDM2 and pro-apoptotic Bcl-2 family protein inhibition.

Notch pathway overactivation is key to T-ALL pathogenesis. *MYC* is among the numerous target genes activated by Notch1, and has been proposed to be one of the most critical for T-ALL growth and maintenance [48–50]. Increased expression of *MYC* has been reported in the majority of T-ALL cases [51]. *FBXW7* mutations are present in ~15% of T-ALL patients [3]. In addition to activating Notch1, they also stabilize Myc protein expression through regulation of its ubiquitination [52]. Activation of the PI3K/AKT pathway is detectable in 70–85% of patients with T-ALL, in roughly 5–10% of cases due to inactivating mutations of *PTEN* [53, 54]. A recent study showed that activation of the PI3K/AKT pathway activates Myc signaling by stabilizing Myc protein expression [51].

Myc is a master transcription factor that regulates up to 15% of genes, including many involved in proliferation and metabolism [55]. However, Myc expression by itself is not sufficient to induce leukemia/lymphoma [11, 56]. Myc, while inducing cellular proliferation, also induces oncogenic stress, resulting in apoptosis [57]. *TP53* is rarely mutated in T-ALL, even in relapsed patients [12, 13]. Here, we demonstrated that derepression of p53 activity through the prevention of MDM2-p53 association with the second-generation MDM2 inhibitor idasanutlin has modest p53-dependent activity against T-ALL PDX as a single agent therapy.

Increased Myc expression can induce oncogenic stress though multiple mechanisms, including induction of DNA replicative stress and ribosome biogenesis stress [52, 58]. Whether these pathways are activated, and to what degree, in T-ALL is an important area of future investigation. Moreover, studies to further define the relative contributions of different BH3 domain proteins in the regulation of apoptosis in T-ALL are worthwhile, since they may help optimize anti-BH3 domain therapy in this cancer.

Given the evidence of upregulation of pro-apoptotic proteins in response to idasanutlin exposure, we explored a rational combination of idasanutlin and navitoclax, a Bcl-2/Bcl-xL inhibitor. We observed strong p53-dependent synergistic activity, and induction of apoptosis by idasanutlin and navitoclax in vitro against a panel of T-ALL PDX lines. RNA sequencing of treated PDX cells was consistent with a convergence on apoptosis, with idasanutlin inducing gene expression level upregulation of the p53 pathway and navitoclax leading to minimal additional RNA level changes. The addition of navitoclax to idasanutlin in combination therapy was associated with a significant increase in NF-*κ*B pathway gene expression. Whether NF-*κ*B signaling directly contributes to induction of apoptosis in this setting will require further study.

We demonstrated that idasanutlin and navitoclax have strong activity in vivo in human T-ALL xenotransplantation models. Combination therapy demonstrated synergistic activity in suppression of tumor burden and increased survival in a panel of four T-ALL PDX. Hence, the present work provides strong support for therapeutic efficacy of simultaneous MDM2 inhibition and Bcl-2 family protein inhibition against T-ALL. There is concern that the dose-limiting thrombocytopenia observed with navitoclax treatment due to on-target Bcl-xL inhibition in circulating platelets may limit its use in the clinic [17, 59, 60]. Venetoclax, which does not target Bcl-xL, is more widely used in cancer treatment at present. An ongoing clinical trial evaluating the combination of both venetoclax and navitoclax in relapsed ALL, including T-ALL, is underway (NCT05192889) and should provide data on safety and tolerability. Additionally, there is an ongoing trial of idasanutlin in combination with venetoclax in patients under 30 years of age with refractory/relapsed neuroblastoma, r/r AML, and r/r ALL (NCT04029688), although T-ALL is a specific exclusion criterion for this study. However, a prior study showed that navitoclax has superior activity in T-ALL compared to venetoclax [31]. We observed that the combination of navitoclax and idasanutlin is superior to venetoclax and idasanutlin, providing more potent and consistent synergistic killing. All three agents, navitoclax, venetoclax, and idasanutlin, share some overlap in safety profile including gastrointestinal toxicity and myelosuppression [16, 29, 34]. Of note, the synergistic response of T-ALL to navitoclax and idasanutlin may permit the use of lower, more tolerable, doses of navitoclax while maintaining on-target Bcl-xL inhibition.

A limitation of this study remains that in vitro only five PDX lines were assessed, and in vivo only PDX lines from four different patients were investigated. Although our findings were robust across these lines, they cannot represent the complete diversity of all T-ALL mutational profiles. However, the PDX lines selected are broadly representative, harboring a combination of frequent *NOTCH1* and/or *FBXW7* mutations, biallelic *CDKN2A* loss, and wildtype *TP53*; all T-ALL genetic hallmarks. These lines are representative and generalizable to T-ALL genomics across a majority of cases. Acknowledging this limitation, the strong and robust response across these lines, which carry commonly recurring T-ALL mutations, suggests that human trials for idasanutlin and navitoclax combination therapy in wildtype *TP53* cases are merited.

### Supplementary information

Supplemental Materials and Methods

Supplemental Figures

Table S1

## Data Availability

All datasets generated during the current study are available from the corresponding author upon reasonable request. Raw reads and processed RNA-seq gene counts generated for this study are available at Gene Expression Omnibus accession number GSE240444.
