# Supplementary material for: Idasanutlin and navitoclax induce synergistic apoptotic cell death in T-cell acute lymphoblastic leukemia
Source: Leukemia. 2023 Oct 14;37(12):2356–66. doi: 10.1038/s41375-023-02057-x (PMC10681904; doi:10.1038/s41375-023-02057-x)
Supplement: Supplementary file 1 — Supplemental Materials and Methods [file 41375_2023_2057_MOESM1_ESM.pdf]

Supplemental Materials and Methods

Quantitative Real-Time PCR

RNA was isolated in 1 mL of TRIzol (Invitrogen) and extracted following the manufacturer's instructions and complementary DNA was prepared using a Reverse Synthesis Kit (Bio-Rad iScript Supermix). Quantitative Real-Time PCR (qRT-PCR) was performed using the TaqMan Universal RT Master Mix (Applied Biosystems). Data were collected on a StepOnePlus Real-Time PCR System (Applied Biosystems). Exon-spanning TaqMan probe primers were from Integrated DNA Technologies, BBC3/Puma FAM (Hs.PT.58.39966045), BAX FAM (Hs.PT.56a.19141193.g) CDKN1A/p21 FAM (Hs.PT.58.40874346.g); and Thermo Fisher Scientific: GAPDH VIC (Hs 02758991_g1).

Immunoblotting

T-ALL cells were lysed with RIPA buffer (Cell Signaling) and protease inhibitor cocktail (Fisher) per the manufacturer's instructions, and proteins were denatured in SDS sample buffer with 9% β-mercaptoethanol at 100°C for 5 minutes. Following BCA protein assay quantification (Lamda Biotech), lysates were reduced with β-mercaptoethanol and boiled prior to electrophoretic separation using a 4-12% Bis-Tris gel (Invitrogen). Proteins were transferred onto a PVDF membrane (Bio-Rad), blocked with 5% milk and blotted for p53 (BioLegend 645802), Bax (Cell Signaling 5023S), Puma (Cell Signaling 98672S), p21 (Cell Signaling 2947S) or β-actin (Sigma-Aldrich A5441). Bands were visualized using host species-specific secondary antibodies conjugated to HRP and detected with high-sensitivity HRP substrate (Thermo Fisher). Blots were visualized with the ChemiDox XRS System (Bio-Rad).

Caspase-3/7 Activity and CellTiter Viability Assays

Caspase-3/7 Activity was assessed with the Caspase-Glo 3/7 Assay (Fisher PRG8091). Per manufacturer's instructions, consistent numbers of cells were plated in triplicate and treated with control dimethyl sulfoxide (DMSO) or idasanutlin. Resuspended cell cultures were pulled in replicate for measurement of viability at 0, 4, 24, and 48 hours. Viable cell number was assessed with the CellTiter-Glo 2.0 Cell Viability Assay (Fisher PRG9242). Per manufacturer's instructions, consistent numbers of cells were plated in triplicate and treated with control DMSO, idasanutlin, or navitoclax. Resuspended cell cultures were pulled in replicate for measurement of viability at 0, 24, 48, and 72 hours. Plates were read on the Synergy H1 Hybrid Reader (BioTek 8041000).

Flow Cytometry

Peripheral blood was collected via facial bleeding and red blood cells lysed in ACK lysis buffer, resuspended in phosphate-buffered saline (PBS) containing 0.1% bovine serum albumin with Fc-block reagent (Trustain FcX; BioLegend), and incubated with antibodies on ice for 20 minutes. After washing, cells were resuspended in SYTOX Red (Invitrogen) for live/dead discrimination. Leukemic cells were defined by human CD45-PE positivity (Fisher BDB555483). Data were collected on an Attune NxT Flow Cytometer and analyzed using FlowJo v10. Apoptosis assays in the T-ALL cultures followed 48 hours of treatment with control DMSO, idasanutlin, or navitoclax, which were resuspended in DMSO and used at the specified final concentrations. Cells were washed

in EDTA-free media and resuspended in Annexin V-PE (BioLegend 640947), according to the manufacturer's instructions. For cell cycle analysis, cells were fixed with BD Cytofix/Cytoperm solution (BD Biosciences BDB554714) and stained with anti-Ki67-AF700 antibody, washed, and resuspended in FxCycle Violet (Invitrogen F10347).

RNA Sequencing

MOLT-3 clones were treated with DMSO, idasanutlin, navitoclax, or combination for 16 hours before RNA was purified using the Monarch Total RNA Miniprep Kit (NEB T2010S). Total RNA integrity was determined using Agilent Bioanalyzer or 4200 Tapestation. Library preparation was performed with 500ng to 1ug of total RNA. Ribosomal RNA was blocked using FastSelect reagents (Qiagen 334385) during cDNA synthesis. RNA was fragmented in reverse transcriptase buffer with FastSelect reagent and heating to 94 degrees for 5 minutes, 75 degrees for 2 minutes, 70 degrees for 2 minutes, 65 degrees for 2 minutes, 60 degrees for 2 minutes, 55 degrees for 2 minutes, 37 degrees for 5 minutes, 25 degrees for 5 minutes. mRNA was reverse transcribed to yield cDNA using SuperScript III RT enzyme (Life Technologies, per manufacturer's instructions) and random hexamers. A second strand reaction was performed to yield ds-cDNA. cDNA was blunt ended, had an A base added to the 3' ends, and then had Illumina sequencing adapters ligated to the ends. Ligated fragments were then amplified for 15 cycles using primers incorporating unique dual index tags. Fragments were sequenced on an Illumina NovaSeq-6000 using paired end reads extending 150 bases. RNA-seq reads were then aligned and quantitated to the Ensembl release 101 primary assembly with an Illumina DRAGEN Bio-IT on-premise server running version 4.0.3-8 software.

Gene counts were processed using the R/Bioconductor package EdgeR, with TMM normalization. Weighted likelihoods based on the observed mean-variance relationship of every gene and sample were calculated using the R/Bioconductor package Limma and transformed to moderated log 2 counts-per-million with Limma's voomWithQualityWeights. Differential expression analysis was performed to analyze for differences between conditions and the results were filtered for only those genes with Benjamini-Hochberg false-discovery rate adjusted p-values less than or equal to 0.05. Global perturbations in pathways were detected using the R/Bioconductor package GAGE to test for changes in expression of the reported log 2 fold-changes reported by Limma in each term versus the background log 2 fold-changes of all genes found outside the respective term.

In vivo *PDX model*

All animal studies were approved by the Institutional Animal Care and Use Committee at Washington University in St. Louis under protocol number 20-0495. Samples were prepared according to library kit manufacturer's protocol, indexed, pooled, and sequenced on an Illumina NovaSeq 6000. Basecalls and demultiplexing were performed with Illumina's bcl2fastq2 software. RNA-seq reads were then aligned and quantitated to the Ensembl release 101 primary assembly with an Illumina DRAGEN Bio-IT on-premise server running version 3.9.3-8 software.

Equal numbers of male and female NOD/SCID/IL-2rgamma null (NSG) mice (6-12 weeks of age) were transplanted with PDX cells via intravenous tail vein injection (1×10^6 cells/100 μL PBS). DFCI12, DFCI15, DFAT28537, and CBAT27299 PDX cells were used, the details of which are presented in Table 1. The mice were monitored routinely for evidence of leukemia. Engraftment was assessed via flow cytometry of peripheral blood mononuclear cells for human CD45 (see

“Flow Cytometry”). Peripheral blood samples were monitored for leukemia burden via hCD45 flow cytometry and CBC (Drew Scientific Hemavet 950) weekly throughout and following treatment. Investigator was not blinded to group allocation, however, samples were processed uniformly in bulk and the same flow cytometric gating strategy was applied across all samples. Synergic drug activity was assessed in each case based on a modified Bliss Independence test, analyzing average daily change for synergy interaction between the dual therapy and the most effective single agent therapy. Variance in tumor burden was similar between the treatment groups being statistically compared. The survival of mice was tabulated and is presented in Kaplan-Meier curves.
