# Supplementary material for: Idasanutlin and navitoclax induce synergistic apoptotic cell death in T-cell acute lymphoblastic leukemia
Source: Leukemia. 2023 Oct 14;37(12):2356–66. doi: 10.1038/s41375-023-02057-x (PMC10681904; doi:10.1038/s41375-023-02057-x)
Supplement: Supplementary file 2 — Supplemental Figures [file 41375_2023_2057_MOESM2_ESM.pdf]

SUPPLEMENTAL FIGURES

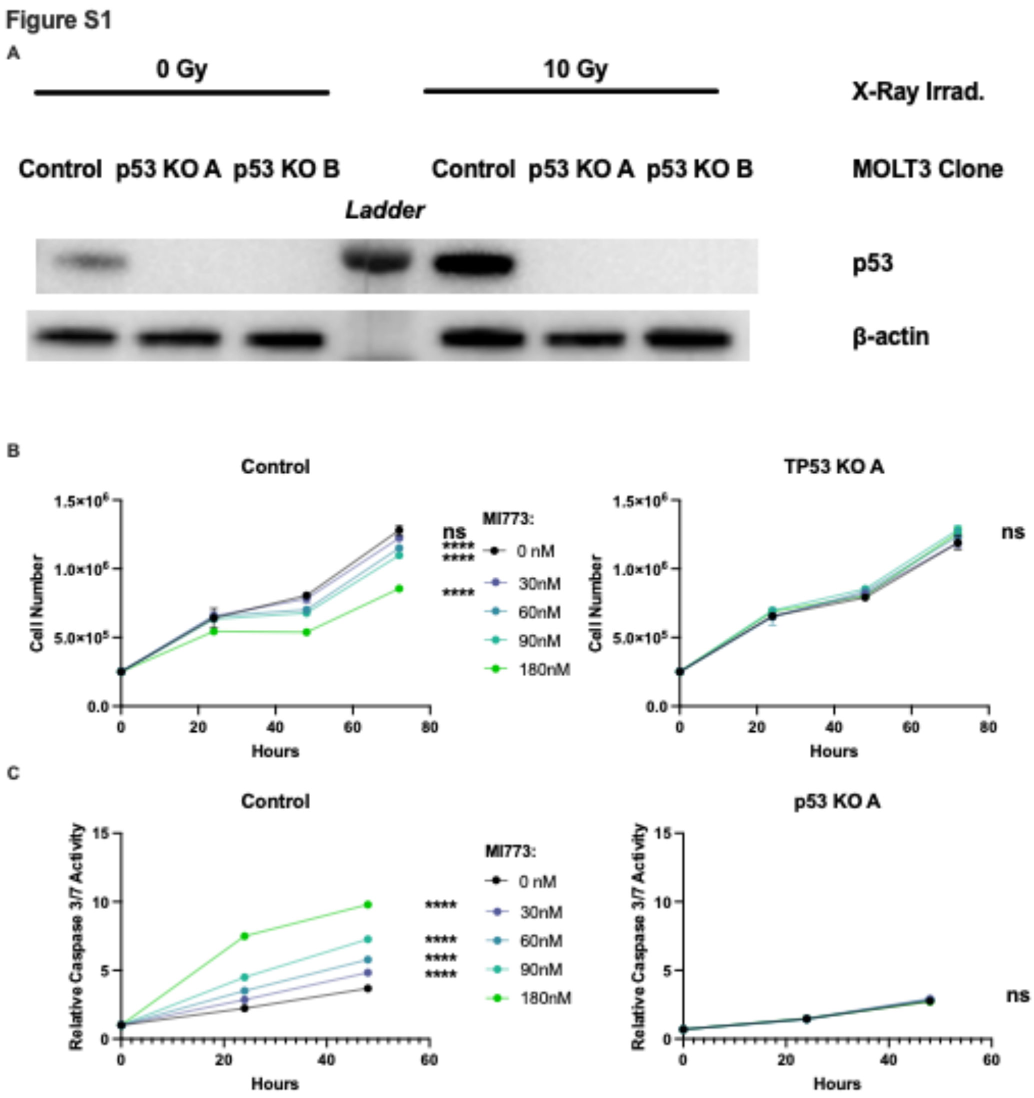

Figure S1: **Supplement to: Idasanutlin has p53-dependent activity against T-ALL immortalized cell lines.** (A) P53 protein expression of AAVS-targeted (control) or *TP53*$^{-/-}$ (p53 KO) MOLT-3 cells 6 hours after x-ray irradiation with 0 or 10 Gray. (B) Control or p53 KO MOLT-3 were treated with the MDM2 inhibitor MI-773 (30, 60, 90, 180nM); cell numbers over 72 hours are shown. (C) Relative caspase-3/7 activity over 48 hours of vehicle or MI-773 treatment. All experiments performed in triplicate. Error bars represent SEM (∗∗∗∗ ≤ 0.0001, ∗∗∗ ≤ 0.001, ∗∗ ≤ 0.01, ∗ ≤ 0.05 compared to DMSO). (B-C) two-way ANOVA.

Figure S2

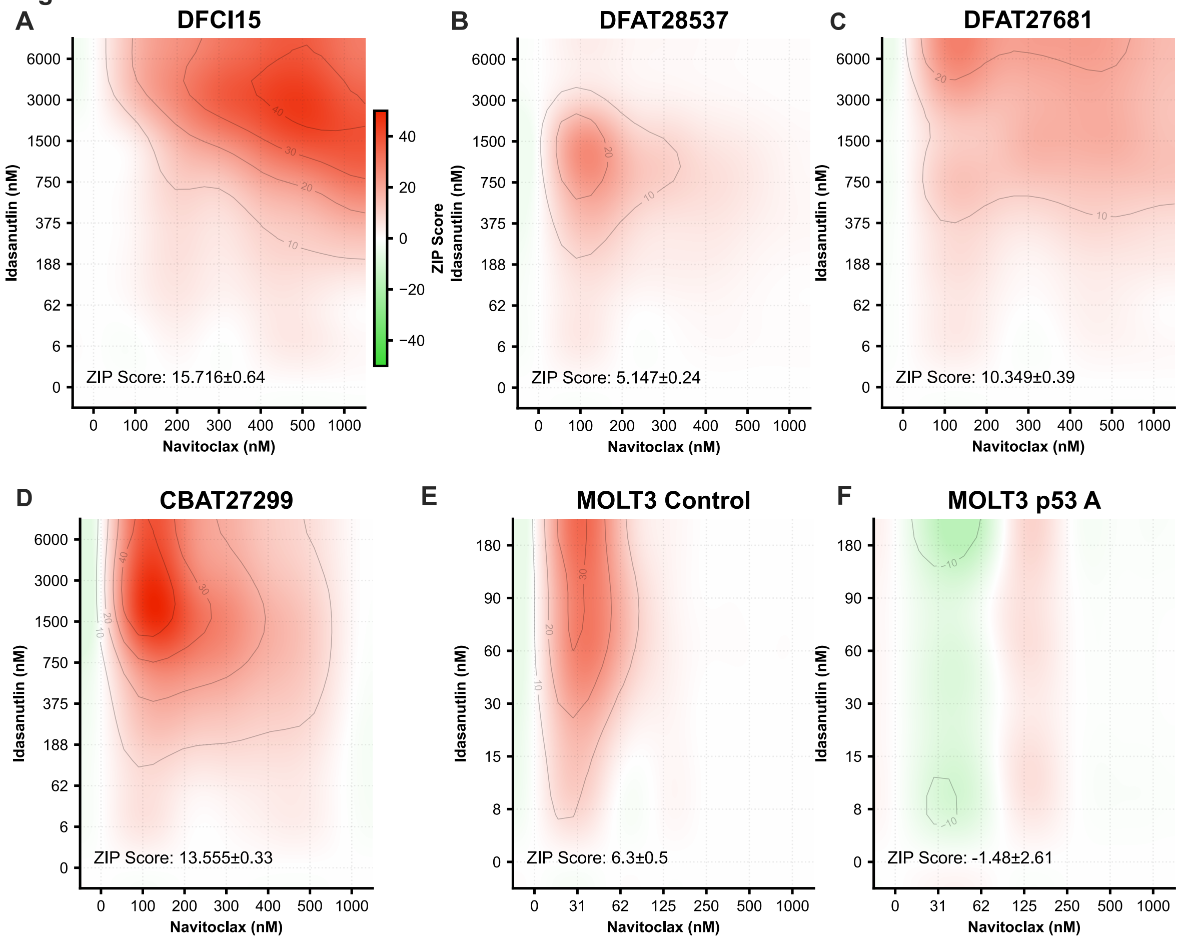

Figure S2: **Navitoclax supplement to: The combination of idasanutlin and navitoclax has synergic activity against T-ALL PDX lines *in vitro*.** (A-D) Representative ZIP synergy plots for DFCI15 (A), DFAT28537 (B), DFAT27681 (C), or CBAT27299 (D) cells based on a dose-response matrix data testing the combination of idasanutlin (vehicle, 6nM, 60nM, 188nM, 375nM, 750nM, 1.5μM, 3μM, 6μM) and navitoclax (vehicle, 100nM, 200nM, 300nM, 400nM, 500nM, 1μM). (E-F) Representative ZIP synergy plots for control (E) or p53 KO MOLT-3 cells (F) treated with idasanutlin (vehicle, 8nM, 15nM, 30nM, 60nM, 90nM, 180nM) and navitoclax (vehicle, 31nM, 62nM, 125nM, 250nM, 500nM, 1μM). All experiments performed in triplicate. (A-F) Zero Interaction Potency Score.

Figure S3

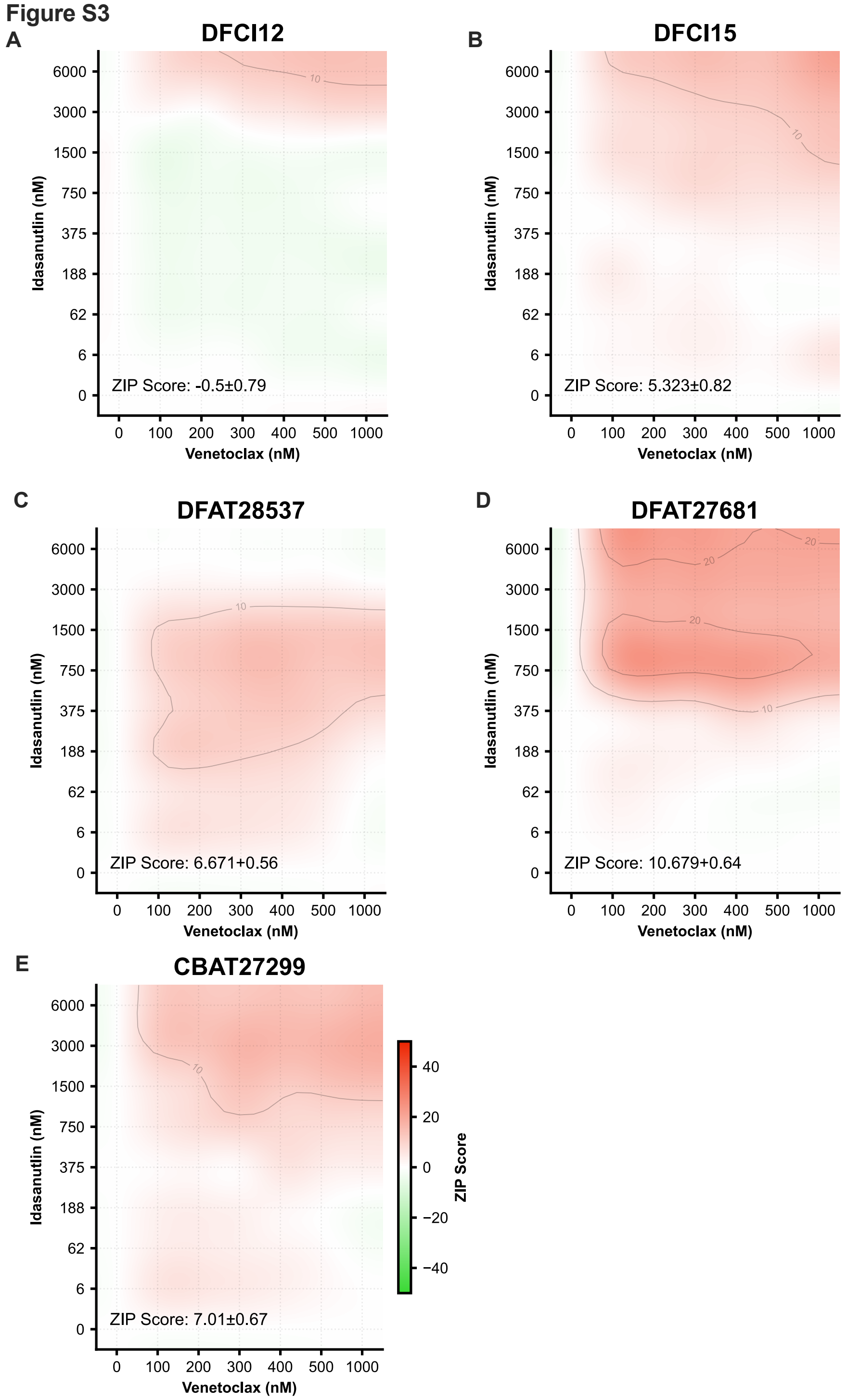

Figure S3: **Venetoclax supplement to: The combination of idasanutlin and navitoclax has synergic activity against T-ALL PDX lines *in vitro*.** (A-E) Representative ZIP synergy plots for DFCI12 (A), DFCI15 (B), DFAT28537 (C), DFAT27681 (D), and CBAT27299 (E) cells based

on a dose-response matrix data testing the combination of idasanutlin (vehicle, 6nM, 60nM, 188nM, 375nM, 750nM, 1.5μM, 3μM, 6μM) and venetoclax (vehicle, 100nM, 200nM, 300nM, 400nM, 500nM, 1μM). All experiments performed in triplicate. (A-E) Zero Interaction Potency Score.

Figure S4

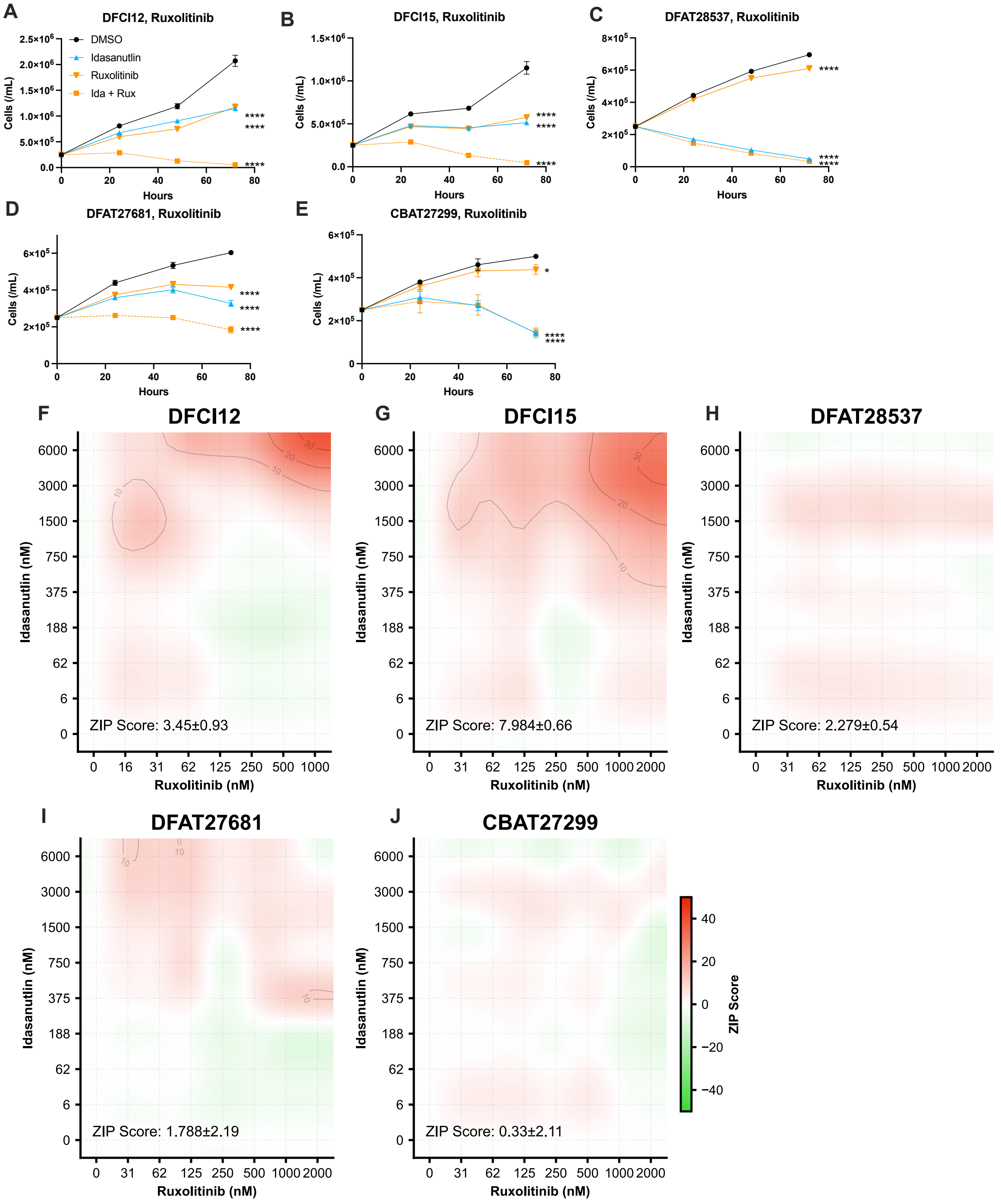

Figure S4: **Ruxolitinib supplement to: The combination of idasanutlin and navitoclax has synergic activity against T-ALL PDX lines *in vitro*.** (A-E) DFCI12 (A), DFCI15 (B), DFAT28537 (C), DFAT27681 (D), and CBAT27299 (E) cells were treated *in vitro* over 72 hours

with vehicle, idasanutlin (1.5μM), ruxolitinib (1μM), or combination therapy and cell number was quantified. (F-J) Representative ZIP synergy plots for DFCI12 (F), DFCI15 (G), DFAT28537 (H), DFAT27681 (I), and CBAT27299 (J) cells based on a dose-response matrix data testing the combination of idasanutlin (vehicle, 6nM, 60nM, 188nM, 375nM, 750nM, 1.5μM, 3μM, 6μM) and ruxolitinib (vehicle, 31.2nM, 62.5nM, 125nM, 250nM, 500nM, 1μM, 2μM). All experiments performed in triplicate. Error bars represent SEM ($**** \leq 0.0001$, $*** \leq 0.001$, $** \leq 0.01$, $* \leq 0.05$ compared to DMSO). (A-E) two-way ANOVA, (F-J) Zero Interaction Potency Score.

Figure S5

A

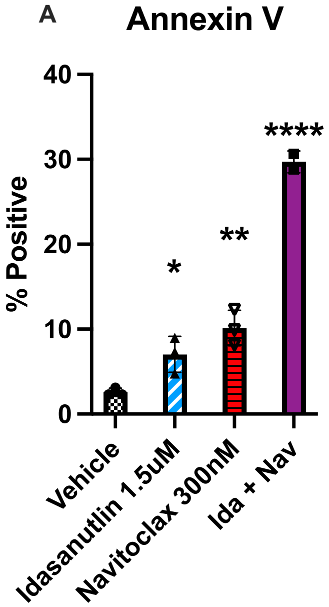

Figure S5: **Supplement to: Transcriptional characterization of treated PDX lines**. (A) DFCI12 cells were treated *in vitro* for 16 hours with vehicle, idasanutlin (1.5μM), navitoclax (300nM), or combination therapy. Panel shows the percentage of apoptotic cells by Annexin V positivity. All experiments performed in triplicate. Error bars represent SEM ($**** \leq 0.0001$, $*** \leq 0.001$, $** \leq 0.01$, $* \leq 0.05$ compared to DMSO). (A) unpaired t-test.
